# Research on a Seepage Monitoring Model of a High Core Rockfill Dam Based on Machine Learning

**DOI:** 10.3390/s18092749

**Published:** 2018-08-21

**Authors:** Xiang Cheng, Qingquan Li, Zhiwei Zhou, Zhixiang Luo, Ming Liu, Lu Liu

**Affiliations:** 1 State Key Laboratory of Information Engineering in Surveying, Mapping and Remote Sensing, Wuhan University, Wuhan 430079, China; xiang.cheng@whu.edu.cn (X.C.); liqq@szu.edu.cn (Q.L.); 2 Key Laboratory of Geo-environmental Surveillance in the Maritime and Marine Zones, National Mapping and Geographic Information Bureau, Shenzhen University, Shenzhen 518061, China; 2172331945@email.szu.edu.cn; 3 State Key Laboratory of Geodesy and Earth’s Dynamics, Institute of Geodesy and Geophysics, Chinese Academy of Sciences, Wuhan 430077, China; 4 Guizhou Water Conservancy and Hydropower Survey and Design Institute, Guiyang 550000, China; liuming930703@163.com (M.L.); liulu196498033@163.com (L.L.)

**Keywords:** high core-wall rockfill dam seepage, abnormal value judgment, principal component analysis, linear regression, osmometer, Nuozhadu, seepage control model

## Abstract

The seepage of a rockfill dam with a high core wall is an important and difficult issue in the safety monitoring of a core rockfill dam, something about which managers are immensely concerned. Seepage of a high core rockfill dam is mainly affected by factors such as water level, rainfall, temperature, filling height, and aging. The traditional research method is to establish a multiple linear regression model to analyze the influence factors of seepage. However, the multicollinearity between these factors affects parameter estimation, and random errors in the data cause the regression model to fail to be established. This paper starts with data collected by an osmometer, uses the 3*δ* criterion to process the outliers in the sample data, uses the R language to perform principal component analysis on the processed data to eliminate the multicollinearity of the factors, and finally uses multiple linear regression to model and analyze the data. Taking the Nuozhadu high core rockfill dam as an example, the influencing factors of seepage in the construction period and the impoundment period were studied and the seepage was then forecasted. This method provides guidance for further studies of the same type of dam seepage monitoring model.

## 1. Introduction

A core-wall rockfill dam is economical to invest in, simple to construct, and locally sourced. It has the advantages of good adaptability to dam foundation conditions, full use of construction excavation materials, and good seismic performance, and it plays an essential role in the development of water resources at home and abroad. It is also a type of earth and rock dam that is widely used in countries all over the world at present [1]. In the 1990s, soil core-wall dams and concrete-face rockfill dams flourished. From the development of core-wall rockfill dams, the construction of high core-wall rockfill dams in China is still in the stage of accumulated experience. The core-wall rockfill dam projects which have been built, such as Lubuge (104 m), Xiaolangdi (160 m), Pubugou (188 m), and Nuozhadu (261.5 m), work well. High core rockfill dams, such as the Lianghekou (295 m) and Shuangjiangkou (314 m) projects currently under construction, are extensive dam projects with a world-class level exceeding 300 m [2].

The seepage monitoring of high core rockfill dams is the focus of safety monitoring, and it is also the issue about which managers are most concerned. Percolation monitoring is mainly accomplished through an osmometer [3,4]. Before automatic monitoring had been implemented, the traditional observation method was to perform a manual reading through the use of secondary instruments [5]. If a record was found to be wrong, the reading would be performed again until the data was recorded in the correct position. After automation, osmometer data is automatically measured by the measurement control unit [6]. Random errors may appear due to loose sensor wiring [7], communication errors [8], hardware failure [9], and so on. The seepage flow is mainly affected by water level, rainfall, temperature, filling elevation, and aging, and it is the main factor that influences seepage during construction and storage periods. The traditional research method has been to establish a multiple linear regression model to analyze the influence factor of seepage [10,11]. However, the calculation of random error participation in a monitoring model will affect the accuracy of the model and the effect of regression. Some studies had mentioned that the abnormal data in the monitoring of bridge structure, the multiple linear regression was failed [12]. At the same time, there are multicollinearities between these impact factors [13]. Therefore, it is necessary to solve the problem of multicollinearity through certain methods [14].

To solve the problems of the traditional methods, this paper first used the 3*δ* criterion to process the outliers in the data collected by the measurement and control unit and then analyzed the principal component of the data without any random errors after processing, thus eliminating the multicollinearity between the factors [15]. Finally, a multivariate linear regression model was established to analyze the relevant influencing factors of seepage from the measured data during the construction period and the impoundment period, from which the key influencing factors for the construction and impoundment periods were obtained [16]. At the same time, the model was used to predict seepage [17,18], the prediction effect of which was found to be good.

The contents of the article are as follows: Section 2 gives the main areas of research and the layout of typical osmometers. Section 3 is a detailed study of the steps and data processing. Section 4 shows in detail the seepage monitoring model and seepage prediction during the construction and storage periods, and Section 5 is the conclusion.

## 2. Research Area 

### 2.1. Introduction of the Nuozhadu High Core-Wall Rockfill Dam

**Figure 1:**
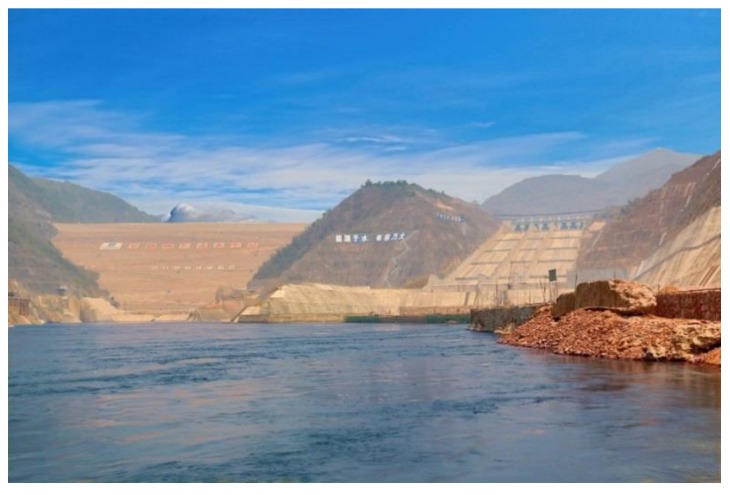
Nuozhadu Hydropower Station.

The Nuozhadu Hydropower Station is located at the lower reaches of the Lancang River at the junction of Cuiyun District and Jixian County, Simao City, Yunnan Province (the dam site is between the Kanjie River and the Burn Village Ditch) It is the fifth level of the eight cascade planning in the middle and lower reaches of the Lancang river. The project is a large (I) first-class project, and the permanent major hydraulic structure is a class 1 building. The project is mainly designed for electricity generation and also has comprehensive utilization benefits such as flood control, irrigation, aquaculture, and tourism. The reservoir has many years of regulating performance. The total storage capacity of the reservoir is 237.03 × 108 m^3^, and the installed capacity of the power station is 5850 MW (9 × 650 MW). The project consists of a core-wall rockfill dam, an open spillway on the left bank, a flood discharge tunnel on the left and right banks, an underground water diversion system on the left bank, a ground 500 kV switch station, and diversion projects. The core-wall rockfill dam is 627.87 m in length at the top, 18 m in width at the crest, 821.5 m in height at the crest, and the largest dam height is 261.5 m. It is currently the third largest in the world and the largest-scale core-wall rockfill dam in Asia [19,20]. The actual map of the Nuozhadu Hydropower Station is shown in Figure 1.

**Figure 2:**
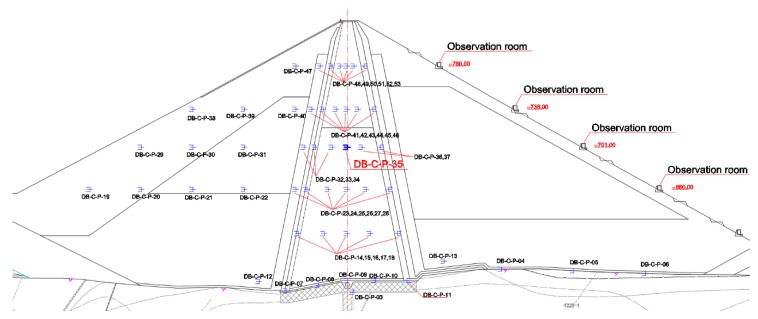
Schematic diagram of osmometer layout of the C section.

In order to effectively monitor the seepage of core-wall rockfill dams, osmometers are embedded in the dam body for monitoring. The osmometers are mainly arranged on the four monitoring sections A, C, D, and E, with 24 of the osmometers in the A section, 53 in the C section, 24 in the D section, and 7 in the E section [21]. The osmotic pressure gauge layout of the C section is shown in Figure 2.

### 2.2. Layout of Typical Osmometer

**Figure 3:**
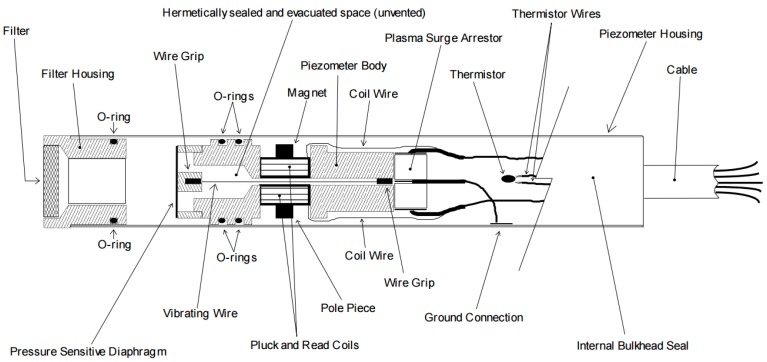
Physical map of vibrating string sensor GK-4500S.

The C-section typical osmometer DB-C-P-35 was chosen as the research object. The osmometer, selected from the American GEOKON vibrating string sensor GK-4500S 3 MPa, is shown in Figure 3.

The main technical indicators are shown in Table 1.

**Figure 4:**
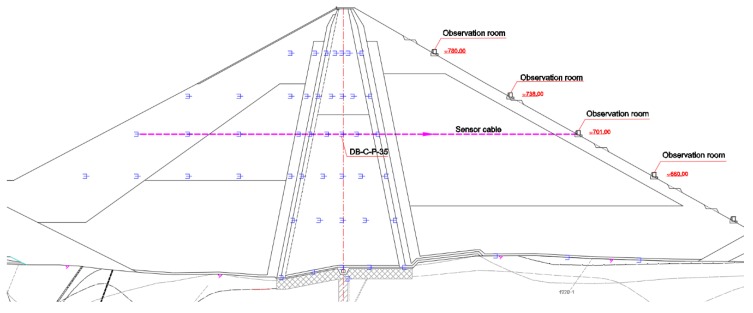
Schematic diagram of burial of typical osmometer (DB-C-P-35) and cable traction.

The osmometer is buried inside the dam body, and the sensor cable is pulled to the observation room outside the nearby dam body. The schematic diagram of burial of typical osmometer (DB-C-P-35) and cable traction is shown in Figure 4.

The osmometer was manually measured by a vibrating wire reader before access automation. Generally, the manual measurement process is as follows: read the osmometer three times, take the average of the three times as the final reading, manually record the results of the three readings and the average value, and fill it in the table. The observation frequency is twice a month during the construction period [22]. After access automation, the osmometer is measured by a high-precision measurement control unit and the observation frequency is once a day [23].

## 3. Study Steps and Processes

The following steps were carried out in this study:

1. Select respectively independent variables and dependent variable data for the construction period and the storage period.
2. Artificially collect sensor data manually to identify errors and reject them. Automated data acquisition uses a 3*δ* criterion to automatically identify errors and reject them.
3. Perform multicollinearity diagnosis on the remaining error-free data in the second step. If there is multicollinearity between the factors, go to the fourth step.
4. Using principal component analysis to eliminate multicollinearity between factors, extract principal components and construct a regression model.
5. Restore the normalized independent variable to the original independent variable to obtain the regression coefficient of the original independent variable.
6. Use the established seepage monitoring model to predict the construction period and the impoundment period, respectively.

**Figure 5:**
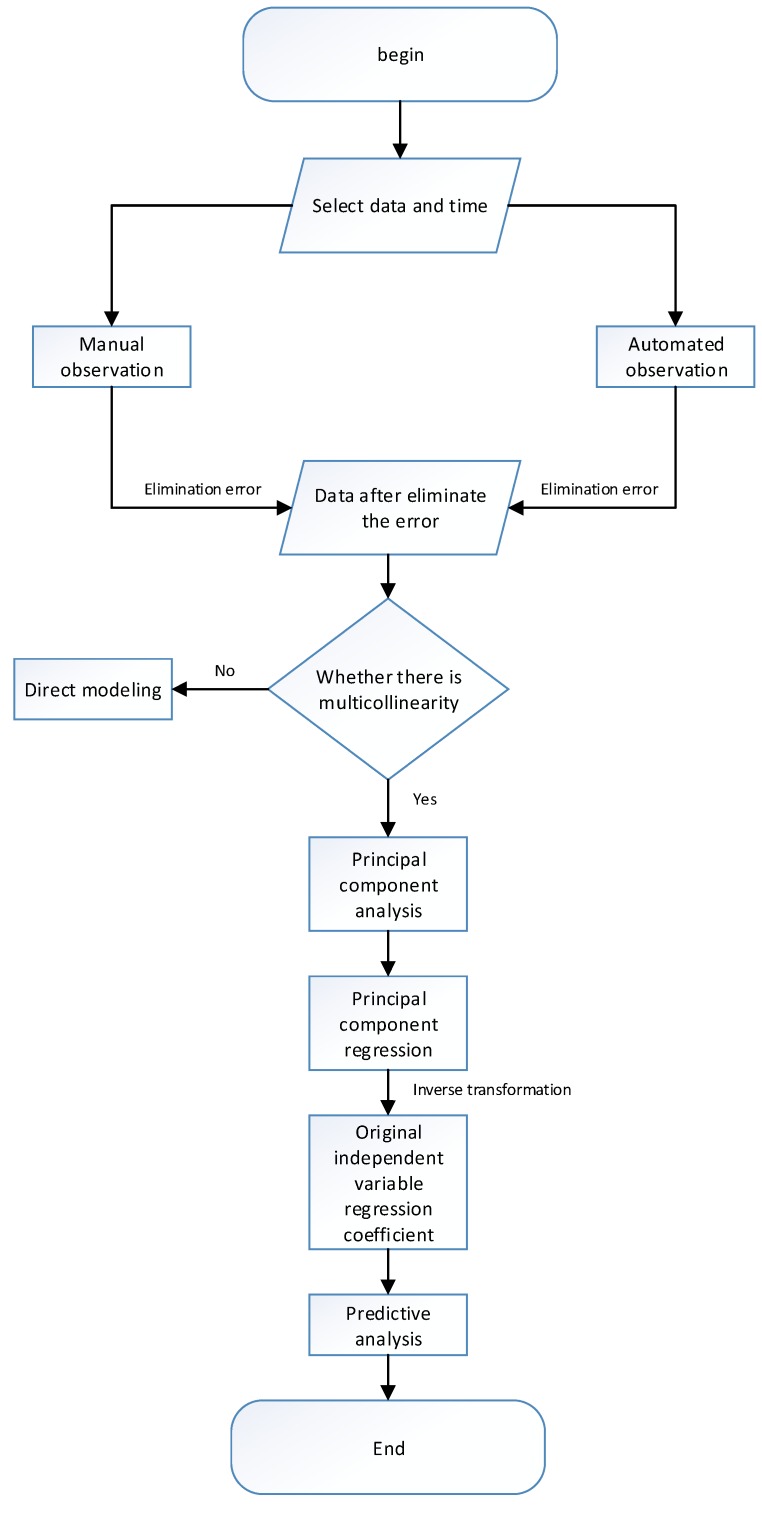
The flow chart of research work.

The main flow chart is shown in Figure 5.

### 3.1. Abnormal Value Judgment

For manually observed osmometer data, if abnormal values are found in the recording process, they can be manually identified and removed to ensure that these abnormal data are not used in the calculation. For the data from automatic monitoring, due to problems such as sensor wiring looseness, interface aging, voltage instability, communication error, and hardware failure in the actual projects, the observation data produces random errors that obey normal distribution [24]. If these abnormal values are directly involved in the establishment of the regression model, the stability and reliability of the model will be affected. In order to eliminate these outliers, the 3*δ* criterion (Puata criterion) was used in this paper [25], the main calculation process is presented below.

The osmotic pressure value measured by the percolometer is recorded as $\left\{y_{1},y_{2},\ldots ,y_{i}\right\}$. Calculate the arithmetic mean $\bar{y}$ and dispersion $V_{i}=y_{i}-\bar{y}(1\leq i\leq n)$. The standard error is calculated according to the Bessel formula [26], as shown in Equation (1):

(1) $$\sigma =\sqrt{\frac{\sum_{i=1}^{n}{\left(y_{i}-\bar{y}\right)}^{2}}{n-1}}$$

If the absolute value of the dispersion is greater than 3 times the standard deviation, which is $\left|V_{i}\right|\geq 3\sigma$, $y_{i}$ can be considered an abnormal value and should be removed.

### 3.2. Principal Component Analysis

The seepage of core-wall rockfill dams is mainly affected by water level, rainfall, temperature, filling elevation, aging, etc. When there is multicollinearity between factors, the least-squares method for regression analysis will be affected, resulting in parameter distortion and thus weakening the model’s predictive function [27]. To judge multicollinearity, this study generally used the variance inflation factor (VIF) [28].

Related studies have proposed ridge regression and principal component analysis to solve the problem of multicollinearity between factors [29]. Ridge regression mainly reduces the mean squared error by ridge parameters, but the selection of ridge parameters has not been completely solved theoretically [30]. This paper mainly solved the problem of multicollinearity through principal component analysis. The basic principle of principal component analysis is to reduce the dimensionality of the original multivariable high-dimensional system and convert multiple indexes into a few comprehensive indicators, which provides a more intuitive understanding of the contribution rate of each independent variable to the dependent variable and ensures that the contained information does not duplicate. The calculation steps of the principal component analysis method are as follows [31]:

1. Standardize the original data. Transform the sample data according to Equations (2) and (3):
  (2) $$Z_{ij}=\frac{x_{ij}-\bar{x_{j}}}{S_{j}}\;i=1,2,\cdots ,n;\;j=1,2,\cdots ,p;\;n>p$$
  and
  (3) $$\bar{x_{j}}=\frac{\sum_{i=1}^{n}x_{ij}}{n};\;S_{j}^{2}=\frac{\sum_{i=1\;}^{n}{\left(x_{ij}-\bar{x_{j}}\right)}^{2}}{n-1}$$
  Among them, $Z_{ij}$ is the standardized data and $X_{ij}$ is the original data.
2. Find the correlation coefficient matrix R for the normalized matrix Z [32].
3. Solve the characteristic equation $\left|R-\lambda I_{p}\right|=0$ (I is the identity matrix) of the correlation matrix *R* to get *P* eigenvalues. Generally, take the cumulative contribution rate of $\frac{\sum_{j=1}^{m}\lambda j}{\sum_{j=1}^{p}\lambda j}\geq 0.85$ corresponding to the eigenvalues $\lambda_{1},\lambda_{2},\cdots \lambda_{m}$ of 1st, 2nd, …, *M*th principal component.

After principal component analysis, multicollinearity between the factors was eliminated. From the cumulative contribution rate, the principal components were extracted and the principal component regression model was constructed. Finally, the regression coefficient of the original independent variable was obtained by the inverse transformation reduction to the original independent variable.

## 4. Achievement

Experimental data was collected from the typical osmometer DB-C-P-35 at the C section of the Nuozhadu core-wall rockfill dam. The time period was selected from the installation of the sensor to the completion of the dam filling (9 December 2010 to 4 December 2012). The storage period was from 1 July 2013 to 15 September 2013. The method described in this paper was used to establish and predict the seepage model during construction and operation.

### 4.1. Percolation Monitoring Model during Construction

The osmotic pressure inside the core during the construction period is mainly affected by factors such as dam filling, temperature, rainfall, and time. During the construction period of the Nuozhadu core-wall rockfill dam, the osmometers were all observed artificially and errors were identified and eliminated artificially while valuing. The regression model between the establishment of osmotic pressure and each influencing factor is shown in Equation (4):

(4) $$\delta_{P}=\delta_{H}+\delta_{T}+\delta_{T^{\prime }\;}+\delta_{J}$$

$\delta_{P}$ is the osmotic pressure, $\delta_{H}$ is the impact factor of the filling elevation, $\delta_{T}$ is the temperature influence factor, $\delta_{J}$ is the influence factor of rainfall, and $\delta_{T^{\prime }}$ is the time impact shadow.

A detailed decomposition was performed using Equations (4) and (5):

(5) $$P_{\left(n\right)}=\alpha_{0}*(H_{\left(n\right)}-H_{0})+\alpha_{1}*(T_{\left(n\right)}-\bar{T})+\alpha_{2}*In({T^{\prime }}_{\left(n\right)}-{T^{\prime }}_{0}+1)+\alpha_{3}*J_{\left(n\right)}/1000+\alpha_{4}\;$$

In this formula, $P_{\left(n\right)}$ is the osmotic pressure at time n, unit: Mpa; $H_{\left(n\right)}$ is the filling height of core wall at time *n*; $H_{0}$ is the elevation of the sensor, unit: m; $T_{\left(n\right)}$ is the temperature at time *n*; $\bar{T}$ is the average temperature, unit: °C; ${T^{\prime }}_{\left(n\right)}$ is the current time; ${T^{\prime }}_{0}$ is the time for the sensor to be buried; $J_{\left(n\right)}$ is the amount of rainfall at time *n*, unit: m; and $\alpha_{0}$, $\alpha_{1}$, $\alpha_{2}$, $\alpha_{3}$, $\alpha_{4}$ is the undetermined coefficient.

The data of the construction period was selected and the error value was manually subtracted and substituted into Equation (6). The variance inflation factor VIF = 11.225 > 10 was solved and the multicollinearity between factors was found. As shown the flow chart of research work in Figure 5, the first step is analyzing the principal components. Using the function *prcomp* in the R language to calculate cumulative contribution rate of the first three principal components was 98.85%. So the three principal components were extracted and established to principal component regression, which were

(6) $$\left\{ \begin{array}{l} Z_{1}=0.641*X_{1}+0.355*X_{2}+0.550*X_{3}+0.399*X_{4}\\ Z_{2}=-0.219*X_{1}+0.663*X_{2}-0.525*X_{3}+0.486*X_{4}\\ Z_{3}=-0.255*X_{1}-0.569*X_{2}+0.105*X_{3}+0.774*X_{4} \end{array}\;\right.$$

From Equation (6), it can be seen that the first principal components $Z_{1}$ and $X_{1}$, $X_{3}$ have a positive correlation. At the same time, it can be seen from the process line that the osmotic pressure increases with the increase of time and filling elevation.

**Figure 6:**
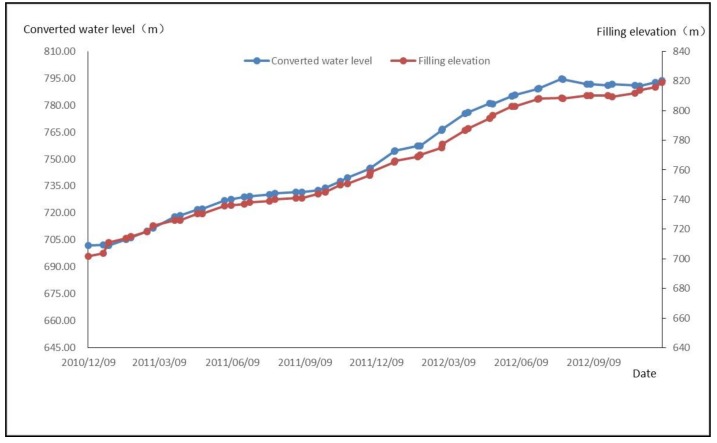
Osmotic Pressure and Filling Elevation Timing Process Diagram.

The process line is shown in Figure 6.

The coefficients in the Equation (6) are after normalization. If we want to solve the coefficients before standardization, it should carry out inverse transformation using the function *apply* in the R language.

Then, using inverse transformation to restore the original argument, the coefficient of the regression equation was

$$\alpha_{0}=0.004,\;\alpha_{1}=0.287,\;\alpha_{2}=0.304,\;\alpha_{3}=-3.360,\;\alpha_{4}=0.387$$

So, the regression equation of the construction period was

(7) $$P_{\left(n\right)}=0.004*(H_{\left(n\right)}-H_{0})+0.287*(T_{\left(n\right)}-\bar{T})+0.304*In({T^{\prime }}_{\left(n\right)}-{T^{\prime }}_{0}+1)-3.360*J_{\left(n\right)}/1000+0.387$$

### 4.2. Seepage Monitoring Model in Water-Storage Period

During the impoundment period, since no change occurs in the filling elevation, the pressure in the core wall is mainly affected by factors such as upstream water level, temperature, rainfall, and time. The dam safety monitoring automation system was implemented during the impoundment period. The data of the osmometer is automatically collected by the measurement control unit (MCU). During the collection process, problems such as loose sensor wiring, aging of the interface, voltage instability, and hardware failure may cause random errors in the observed data. The 3*δ* criterion was used to automatically eliminate the error, and the regression model between the osmotic pressure and the influencing factors was established for the data without measuring errors, as shown in Equation (8):

(8) $$\delta_{P}=\delta_{H}+\delta_{T}+\delta_{T^{\prime }\;}+\delta_{J}$$

$\delta_{P}$ is the osmotic pressure, $\delta_{H}$ is the impact factor of upstream water level, $\delta_{T}$ is the temperature influence factor, $\delta_{J}$ is the influence factor of rainfall, and $\delta_{T^{\prime }}$ is the time impact factor.

A detailed decomposition was performed using Equations (8) and (9):

(9) $$P_{\left(n\right)}=\alpha_{0}*(H_{\left(n\right)}-H_{0})+\alpha_{1}*(T_{\left(n\right)}-\bar{T})+\alpha_{2}*In({T^{\prime }}_{\left(n\right)}-{T^{\prime }}_{0}+1)+\alpha_{3}*J(n)/1000+\alpha_{4}$$

In this formula, $P_{\left(n\right)}$ is the osmotic pressure at time n, unit: Mpa; $H_{\left(n\right)}$ is the upstream reservoir level at time *n*; $H_{0}$ is the elevation of the sensor, unit: m; $T_{\left(n\right)}$ is the temperature at time *n*; $\bar{T}$ is the average temperature, unit: °C; ${T^{\prime }}_{\left(n\right)}$ is the current time; ${T^{\prime }}_{0}$ is the time for the sensor to be buried; $J_{\left(n\right)}$ is the amount of rainfall at time *n*, unit: m; and $\alpha_{0}$, $\alpha_{1}$, $\alpha_{2}$, $\alpha_{3}$, $\alpha_{4}$ is the undetermined coefficient.

The variance inflation factor VIF = 79.865 > 10 was solved and the multicollinearity between factors was found. As shown the flow chart of research work in Figure 5, the first step is analyzing the principal components. Using the function *prcomp* in the R language to calculate cumulative contribution rate of the first three principal components was 98.59%. So the three principal components were extracted and established to principal component regression, which were

(10) $$\left\{ \begin{array}{l} Z_{1}=0.607*X_{1}+0.512*X_{2}+0.602*X_{3}+0.058*X_{4}\\ Z_{2}=-0.030*X_{1}-0.000*X_{2}-0.065*X_{3}+0.997*X_{4}\\ Z_{3}=-0.343*X_{1}+0.857*X_{2}-0.383*X_{3}-0.034*X_{4} \end{array}\;\right.$$

From Equation (10), it can be seen that the first principal components $Z_{1}$ and $X_{1}$, $X_{3}$ have a positive correlation. At the same time, it can be seen from the process line that the water level converted by osmotic pressure rises with the increase of time and the elevation of upstream reservoir water level.

**Figure 7:**
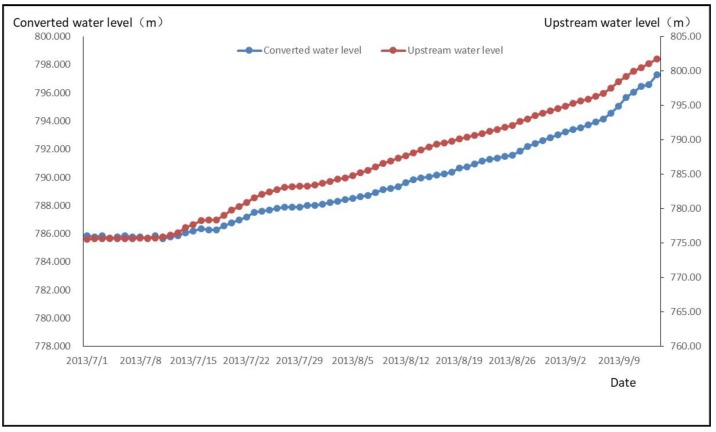
Osmotic Pressure and Upstream Reservoir Level Timing Process Diagram.

The process line is shown in Figure 7.

The coefficients in the Equation (10) are after normalization. If we want to solve the coefficients before standardization, it should carry out inverse transformation using the function *apply* in the R language.

Then, using inverse transformation to restore the original argument, the coefficient of the regression equation was

$$\alpha_{0}=0.002,\;\alpha_{1}=-0.015,\;\alpha_{2}=1.613,\;\alpha_{3}=-0.043,\;\alpha_{4}=-4.138$$

So, the regression equation of the water-storage period was

(11) $$P_{\left(n\right)}=0.002*(H_{\left(n\right)}-H_{0})-0.015*(T_{\left(n\right)}-\bar{T})+1.613*In({T^{\prime }}_{\left(n\right)}-{T^{\prime }}_{0}+1)-0.043*J_{\left(n\right)}/1000-4.138$$

#### Comparison between Traditional Method and Recommended Method

The traditional research method had been to directly establish a multiple linear regression model to analyze the influence factor of seepage, not considering whether there are outliers in the sample data and multicollinearities between seepage impact factors.

A multiple linear regression model was established using Equation (9) and the water-storage period data in the traditional research method through SPSS tools. The result of the analysis was shown in Table 2.

So, the regression equation was

(12) $$P_{\left(n\right)}=0.089*(H_{\left(n\right)}-H_{0})-0.093*(T_{\left(n\right)}-\bar{T})-61.811*In({T^{\prime }}_{\left(n\right)}-{T^{\prime }}_{0}+1)-1.294*J_{\left(n\right)}/1000+178.103\;$$

From Equation (12), it can be seen that the coefficient of time (where it is −61.811) is a negative number. However, the coefficient of time (where it is 1.613) in Equation (11) is a positive one. Actually, the water level rises with the increase of time and the coefficient of time is always positive.

At the same time, it can be seen from Table 2 that the variance inflation factor (VIF) > 10 and there are the multicollinearity among factors. In contrast, there are no multicollinearities among factors in the recommended method because multicollinearities had been eliminated.

**Figure 8:**
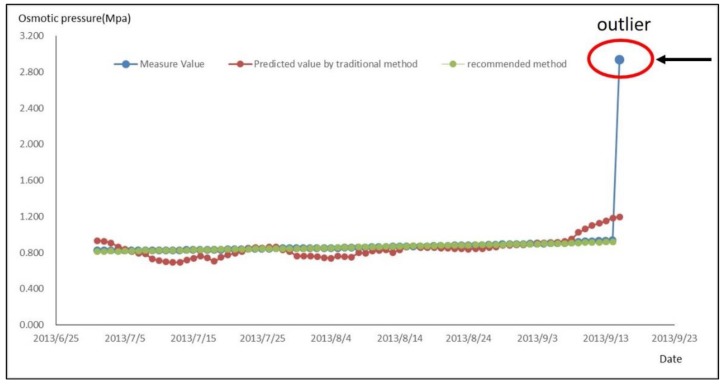
The Measure and Predicted Process Chart of Osmotic Pressure In Traditional and Recommended Method During Water-Storage Period.

In the traditional research method, the data of the dependent variable during the water-storage period was substituted into Equation (12) to calculate the predicted value of the osmotic pressure. The time series of the predicted values in traditional and recommended method and measured values was then plotted, as shown in Figure 8.

Note *R_a_*^2^ as a goodness of fit [33] for the traditional method and *R_b_*^2^ for the recommended method.

*R_a_*^2^ = 0.19 and *R_b_*^2^ = 0.97 were calculated respectively indicating that the recommended method is prior than the traditional method.

### 4.3. Percolation Prediction

**Figure 9:**
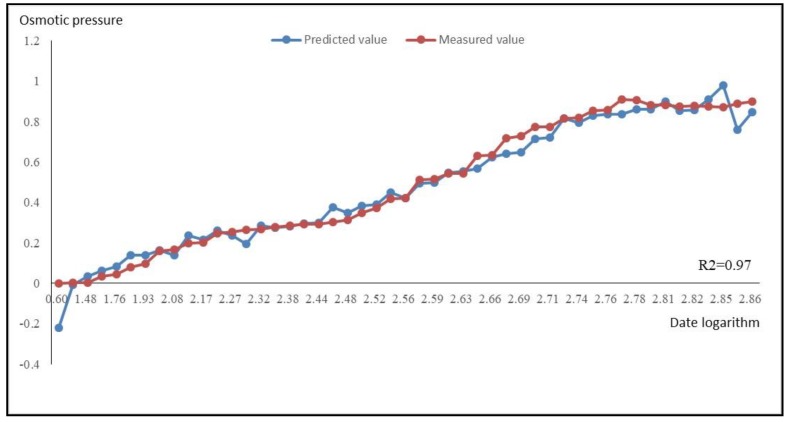
Osmotic Pressure Measured and Predicted Value Process Chart during Construction Period.

The data of the dependent variable during the construction period was substituted into Equation (7) to calculate the predicted value of the osmotic pressure, and the time series of the predicted and measured values was then plotted, as shown in Figure 9.

A goodness of fit [33] of *R*^2^ = 0.97 indicates that the fitting effect is good and the regression equation is suitably predictive.

**Figure 10:**
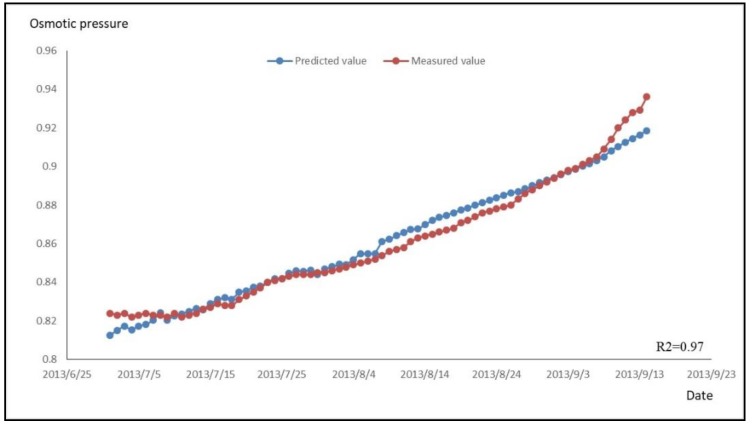
Osmotic Pressure Measured and Predicted Value Process Chart during Water-Storage Period.

Similarly, the data of the dependent variable during the water-storage period was substituted into Equation (11) to calculate the predicted value of the osmotic pressure, and the time series of the predicted and measured values was then plotted, as shown in Figure 10.

A goodness of fit of *R*^2^ = 0.97 indicates that the fitting effect is good and that the regression equation can be used to predict the osmotic pressure during the water-storage period.

## 5. Discussions and Conclusions

Seepage of a high core rock fill dam is mainly affected by factors such as water level, rainfall, temperature, filling height, and aging. The monitoring of seepage is done by vibrating wire osmometer. In order to make the research more universal, the monitoring data of construction period and water storage period are selected to establish the seepage monitoring model in the current research. The recommended method in this paper effectively eliminates the abnormal data collected by the measurement control unit and makes the sample data standard. The principal component analysis is used to solve the problem of the multicollinearity among independent variables, and the influence factors of seepage are correctly analyzed by principal component regression. Meanwhile, using the established model to predict the seepage, the fitting accuracy is high. The traditional multiple linear regression analysis method which is directly established through the sample data, without considering the abnormal values of the sample data and the multicollinearity among the influence factors. So, the established model in traditional method cannot effectively explain the relationship between seepage and influencing variables, and the model has low prediction accuracy or even failed to model.

The recommended method in this paper has established the seepage monitoring model for the construction period and the storage period of a high core-wall rockfill dam and the model was used to predict the seepage flow. The results were shown to be good, which suggests this model should have strong guiding significance for related research on the same type of dam and great practical value in actual project.

There are also some limitations in this paper that the article uses the established regression analysis for the seepage prediction, which needs to know the value of each impact factor and is unable to study autonomously. So, it is a passive prediction method. Further research should be conducted on the seepage prediction method, such as Long Short-Term Memory (LSTM) [34], to establish a deep learning method that can study autonomously, so as to improve the accuracy and efficiency of seepage prediction.

The method of this paper is focused on the measured data of sensors. Actually, the finite element method (FEM) of dam should be built, verifying and contrast with the recommended method in this paper, which is also a further research content in the future.

## Figures and Tables

**Table 1:** Main technical indicators of GK-4500S.

Model	GK-4500S
Standard range	3 MPa
Nonlinearity	straight line: ≤0.5%FS; Polynomial: ≤0.1%FS
Sensitivity	0.025%FS
Overload capacity	50%
Instrument length	133 mm
Outer diameter	19.05 mm

**Table 2:** The result of the analysis in the traditional research method.

Model Parameter	Coefficient	VIF
constant	178.103	
water level	0.089	79.865
temperature	−0.093	1.737
time	−61.811	81.853
rainfall	−1.294	1.009
